# CRISPR/dCas9 Transcriptional Activation of Endogenous Apolipoprotein AI and Paraoxonase 1 in Enterocytes Alleviates Endothelial Cell Dysfunction

**DOI:** 10.3390/biom11121769

**Published:** 2021-11-25

**Authors:** Laura Toma, Teodora Barbălată, Gabriela M. Sanda, Loredan S. Niculescu, Anca V. Sima, Camelia S. Stancu

**Affiliations:** Lipidomics Department, Institute of Cellular Biology and Pathology “Nicolae Simionescu” of the Romanian Academy, 8, B.P. Hasdeu Street, 050568 Bucharest, Romania; laura.toma@icbp.ro (L.T.); teodora.barbalata@icbp.ro (T.B.); gabriela.sanda@icbp.ro (G.M.S.); loredan.niculescu@icbp.ro (L.S.N.); anca.sima@icbp.ro (A.V.S.)

**Keywords:** apolipoprotein AI, endothelial cells, enterocytes, HDL, paraoxonase 1, transcriptional activation

## Abstract

Atherosclerosis is the main cause of cardiovascular diseases with high prevalence worldwide. A promising therapeutic strategy to reverse atherosclerotic process is to improve the athero-protective potential of high-density lipoproteins (HDL). Since the small intestine is a source of HDL, we aimed to activate transcription of the endogenous HDL major proteins, apolipoprotein AI (ApoAI) and paraoxonase 1 (PON1), in enterocytes, and to evaluate their potential to correct the pro-inflammatory status of endothelial cells (EC). Caco-2 enterocytes were transfected with CRISPR activation plasmids targeting ApoAI or PON1, and their gene and protein expression were measured in cells and conditioned medium (CM). ATP binding cassette A1 and G8 transporters (ABCA1, ABCG8), scavenger receptor BI (SR-BI), and transcription regulators peroxisome proliferator-activated receptor γ (PPARγ), liver X receptors (LXRs), and sirtuin-1 (SIRT1) were assessed. Anti-inflammatory effects of CM from transfected enterocytes were estimated through its ability to inhibit tumor necrosis factor α (TNFα) activation of EC. Transcriptional activation of ApoAI or PON1 in enterocytes induces: (i) increase of their gene and protein expression, and secretion in CM; (ii) stimulation of ABCA1/G8 and SR-BI; (iii) upregulation of PPARγ, LXRs, and SIRT1. CM from transfected enterocytes attenuated the TNFα-induced inflammatory and oxidative stress in EC, by decreasing TNF receptor 1, monocyte chemoattractant protein-1, and p22phox. In conclusion, transcriptional activation of endogenous ApoAI or PON1 in enterocytes by CRISPR/dCas9 system is a realistic approach to stimulate biogenesis and function of major HDL proteins which can regulate cholesterol efflux transporters and reduce the inflammatory stress in activated EC.

## 1. Introduction

Cardiovascular diseases (CVD) represent the clinical manifestation of atherosclerosis and are a major threat to public health worldwide, with high prevalence and mortality rates [1,2]. Atherosclerosis develops through structural modifications of the arterial wall that involve lipid accumulation and excessive inflammatory and fibro-proliferative reactions, which in the end lead to the formation of atherosclerotic plaques that can obstruct the blood flow in the vessel [3]. CVD are complex multifactorial diseases, their major risk factors including dyslipidemia, oxidative and inflammatory stress, diabetes, hypertension, smoking, and ageing [1,4]. Endothelial cells (EC) form the inner layer of cells of the arterial wall, and mediate the transport of molecules between plasma and tissues, regulate vascular tone, and synthesize and secrete a variety of factors that control lipid homeostasis, signal transduction, immunity, and inflammation [5]. Due to their location, EC are the first cells of the vessel wall exposed to injurious factors from the blood such as increased lipids and glucose, reactive oxygen species, and pro-inflammatory molecules. Under pathological conditions, alterations in EC function precede the development of atherosclerotic plaques and contribute decisively to the clinical manifestations of CVD [6,7].

High-density lipoproteins (HDL) are macromolecular complexes synthetized by the liver and small intestine, which comprise more than 100 varieties of lipids and over 80 types of proteins with different roles [8]. HDL plays an important role in protection against CVD due to their potential to remove excess cholesterol from the peripheral tissues to the liver for excretion [9], and due to their antioxidant, anti-inflammatory, and anti-thrombotic properties [10]. Epidemiological data show that the risk of CVD is inversely related to the level of serum HDL-cholesterol (HDL-C) and is directly associated with the oxidative and inflammatory stress [10,11]. Nevertheless, several drugs designed to raise HDL-C failed to decrease the incidence of recurrent cardiovascular events in CVD patients [12,13,14]. In addition, it has been demonstrated that CVD may also occur in subjects with normal HDL-C levels, thus suggesting that components of HDL other than cholesterol are more important for HDL function [15]. In the last decade, the hypothesis that HDL function rather than HDL-C levels is more relevant for the status of CVD patients has been generally accepted [10,16,17]. Under normal physiological conditions, HDL protect EC against injurious factors [18]. The ability of HDL to exert protective effects depends on the content and quality of their proteins, such as apolipoprotein AI (ApoAI) and the antioxidant enzyme paraoxonase 1 (PON1) [16,17]. In addition, HDL maturation depends on the ATP binding cassette A1 (ABCA1) transporter, a protein located in the cell membrane, more abundant in enterocytes and hepatocytes, which mediates the lipidation of ApoAI [19]. The gene expression of ApoAI is regulated by the transcription factor peroxisome proliferator-activated receptor γ (PPARγ) [20], while the transcription of ABCA1 is controlled by the liver X receptors (LXRs) [21]. The gene expression of PON1 is regulated by both PPARγ and LXRs [22]. Activation of these transcription factors is, in turn, mediated by other proteins, such as sirtuin 1 (SIRT1), which is an enzyme responsible for the activation by deacetylation of transcription factors, including LXRs and PPARγ [23]. Under pathologic conditions, the levels of ApoAI and PON1 decrease, and they become oxidized or glycated generating dysfunctional HDL [17,24,25].

Numerous therapies have been developed to treat CVD patients, leading to improved life expectancy, but a considerable residual risk of disease remains [1]. Therefore, the development of new therapies to stabilize or reduce atherosclerotic lesions, with minimal side-effects, continues to be a priority. A promising therapeutic strategy is the improvement of HDL function, and several attempts to increase ApoAI levels in CVD patients by intra-venous injection of recombinant human ApoAI, reassembled human ApoAI or ApoAI mimetics have been performed [26]. The benefits for CVD patients have been encouraging, but limited in terms of atheroma regression, while the cost of the therapies was excessive.

The small intestine is a key organ in regulating lipid metabolism, and different anti-atherosclerotic therapies have been developed to target the enterocytes [27,28]. The small intestine is one of the main sources of HDL, but no specific therapy has been developed to stimulate HDL synthesis and secretion by the enterocytes.

In the present study we aim to activate the transcription of endogenous ApoAI and PON1 in human Caco-2 enterocytes by using the CRISPR/dCas9 technology, and to evaluate the potential of the culture medium to correct the pro-inflammatory status of EC activated by tumor necrosis factor α (TNFα).

## 2. Materials and Methods

### 2.1. Reagents

Essential Modified Eagle’s Medium (EMEM), Dulbecco’s Modified Eagle’s Medium (DMEM), streptomycin, penicillin, neomycin, MEM non-essential amino acid solution, L-glutamine, protease inhibitor cocktail, sodium orthovanadate, and sodium fluoride were from Sigma-Aldrich Co., Saint Louis, MO, USA. TNFα (210-TA-020/CF) was from R&D Systems, and ECL chemiluminescent substrate from AppliChem GmbH, Darmstadt, Germany. ApoAI CRISPR activation plasmid (sc-400499-ACT), PON1 CRISPR activation plasmid (sc-402701-ACT), control CRISPR activation plasmid (sc-437275), UltraCruz transfection reagent (sc-395739), and transfection medium (sc-108062) were from Santa Cruz Biotechnology, Santa Cruz, CA, USA. Fetal bovine serum (FBS) was from Euroclone, Milano, Italy. TRIzol reagent, PureLink RNA MiniKit, High capacity reverse transcriptase kit, and SyBr Select Master Mix were from Applied Biosystems, Waltham, MA, USA.

### 2.2. Cell Cultures

Epithelial cells from human colon (Caco-2 cell line) were purchased from ATCC (Manassas, VA, USA). Cells were grown as recommended by the manufacturers, in EMEM supplemented with FBS (10%, *v*/*v*), non-essential amino acid solution, penicillin (100 U/mL), streptomycin (0.1 mg/mL), and L-glutamine (10 mM). Culture medium was changed every 2–3 days until confluency. Caco-2 cells between passages 40 and 60 were used in this study.

Human umbilical vein EC (EA.hy926 line) commercialized by ATCC (Manassas, VA, USA) were cultured in DMEM supplemented with FBS (10% *v*/*v*) in the presence of streptomycin/penicillin (0.1 mg/mL/100 U/mL) and neomycin (50 µg/mL). All cells were incubated at 37 °C in a humidified atmosphere with 95% air and 5% CO_2_.

### 2.3. Overexpression of ApoA-I and PON1 in Caco-2 Cells

**Figure 1:**
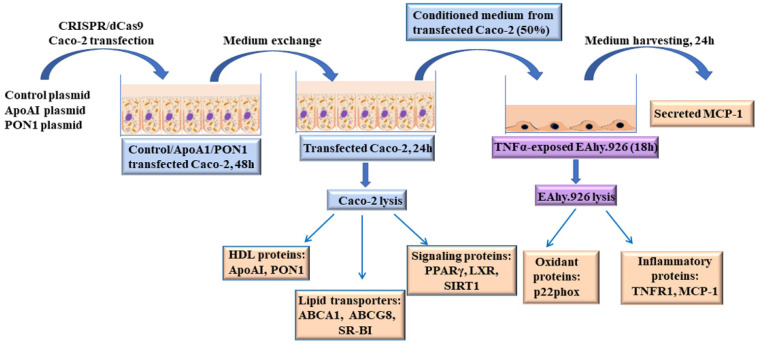
Graphical representation of the experimental design. Caco-2 cells were transfected with ApoAI or PON1 CRISPR activation plasmids. Then, 48 h after transfection, the medium was replaced and the cells were incubated for another 24 h with DMEM to obtain the conditioned media enriched in ApoAI or PONI. After this time interval, Caco-2 cells were lysed and further processed for Real-Time PCR and Western blot analysis. The conditioned media from transfected Caco-2 cells were used to evaluate their effects on EC function.

Caco-2 cells were seeded on 12 well plates in antibiotic free standard growth medium. At 50–80% confluency, cells were transfected with specific commercially available CRISPR activation plasmids for ApoAI (ApoAIp) or PON1 (PON1p), according to manufacturer instructions. Then, 48 h after transfection, the medium was replaced and cells were incubated for another 24 h with DMEM supplemented with 2% FBS, L-glutamine and non-essential amino acids solution, to obtain the conditioned medium (CM) enriched in ApoAI and PONI. After this time interval, Caco-2 cells were lysed and further processed for Real-Time PCR and Western blot analysis. The CM from transfected Caco-2 cells was centrifuged at 400× *g* for 5 min and further used to evaluate their effects on EC function. The experimental design is represented in Figure 1. Caco-2 cells transfected with control CRISPR activation plasmid (Cp) or incubated with transfection media in the absence of activation plasmids were used as negative controls. The concentrations of transfection reagent (5 µL/mL culture medium) and activating plasmids (1 µg plasmid/mL culture medium) for optimal transfection were selected in previous pilot experiments.

### 2.4. Incubation of EC with Conditioned Media from Caco-2 Transfected Cells

The effect of CM from Caco-2 cells overexpressing ApoAI or PON1 was tested on activated EC obtained by exposing the cells to 7.5 ng/mL TNFα for 18 h. After activation, EC medium was replaced and cells were incubated with a mix of CM from transfected Caco-2 cells and DMEM (1:1 ratio), supplemented with D-glucose to a final concentration of 5 mM. After 24 h, the medium from EC was harvested and cells were lysed and processed for Western blot analysis.

### 2.5. Isolation of Total RNA and Quantification of Gene Expression 

After transfection, total RNA was isolated from Caco-2 cells using TRIzol reagent or PureLink RNA MiniKit. A total of 1–2 μg of RNA was reverse transcribed by using the enzyme MultiScribe Reverse Transcriptase, following the manufacturer’s recommendations. The obtained cDNA was amplified using SyBr Select Master Mix and specific primers for ApoAI, PON1, and β-actin (as a housekeeping gene) (Table 1) in a ViiA7 Real-Time PCR system (Applied Biosystems, Waltham, MA, USA). The relative quantification of amplification products was done by the “Fit Point Method” [29] and compared to Cp cells, which were assigned a value of 1.

### 2.6. Quantification of Protein Expression in the Cellular Lysate

Caco-2 cells or EC were washed with cold phosphate buffered saline (PBS) and lysed with RadioImmuno Precipitation Assay (RIPA) buffer enriched with protease and phosphatase inhibitors. In total, 30–50 μg of total cell protein were separated on 10–12% SDS-polyacrylamide gel electrophoresis and transferred to nitrocellulose membranes. After blocking (5% non-fat powered milk for 1 h, at room temperature), the nitrocellulose membranes were probed with specific primary antibodies (Table 2) overnight at 4 °C. The next day, the membranes were incubated with the secondary antibody for 1 h at room temperature and the proteins of interest were detected using ECL chemiluminescent substrate. The relative protein expression (protein of interest to β-actin) was determined by densitometric analysis of the digital image using TotalLab 100 software (Sigma-Aldrich Co., Saint Louis, MO, USA).

### 2.7. Measurement of Secreted Proteins Levels in the Cell Culture Medium

ApoAI, PON1, and monocyte chemoattractant protein 1 (MCP-1) levels were measured in the culture media collected from Caco-2 cells (ApoAI, PON1) or EC (MCP-1). Culture media were slightly centrifuged to eliminate the cell debris, concentrated by using trichloroacetic acid precipitation and processed for Western blot assay as detailed above. The level of the secreted proteins was normalized to the total cell protein and expressed relative to Cp for Caco-2 cells (ApoAI, PON1) and to untreated EC (MCP-1).

### 2.8. Statistical Analysis of the Data

SPSS software v21 (IBM SPSS, IBM Ireland, Dublin, Ireland) was used for statistical analyses. The one-way ANOVA test was used to estimate the statistical differences between the experimental groups. The Mann–Whitney U-test and Independent Student’s T-test were used for the inter-group comparison of the obtained data. *p* values less than 0.05 were considered statistically significant. All data were expressed as mean ± standard error of the mean (SEM). Data from at least three independent experiments were used for the statistical analysis.

## 3. Results

### 3.1. Transcriptional Activation of Endogenous ApoAI by CRISPR/dCas9 Technology Increases ApoAI Expression and Secretion and Stimulates PON-1 Production in Caco-2 Cells

**Figure 2:**
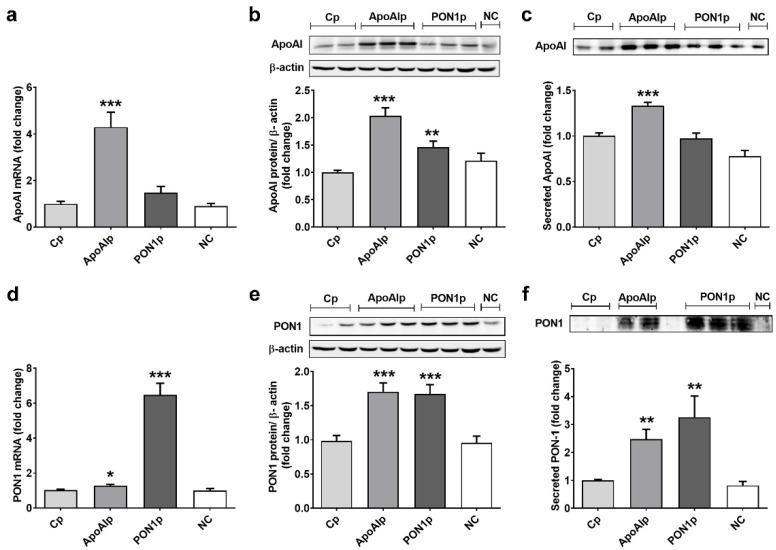
Transcriptional activation of ApoAI and PON1 by CRISPR/dCas9 technology in Caco-2 cells. (**a**,**d**) ApoAI and PON1 mRNA; (**b**,**e**) ApoAI and PON1 protein expression relative to β-actin (representative blot and densitometric analysis); (**c**,**f**) secreted ApoAI and PON1 in the culture medium relative to total cell protein (representative blots and densitometric analysis). Cp, control CRISPR activation plasmid; ApoAIp, CRISPR activation plasmids for ApoAI; PON1p, CRISPR activation plasmids for PON1; NC, negative control–untransfected Caco-2 cells. All data are expressed as fold change versus Cp and presented as mean ± SEM of 3–5 independent experiments. * *p* < 0.05, ** *p* < 0.01, *** *p* < 0.001 vs. Cp.

To assess whether transfection of CRISPR/dCas9 ApoAI plasmids increases the level of ApoAI in Caco-2 cells, the gene and protein expression of this protein was evaluated by Real-Time PCR and Western blot. The results showed that ApoAI gene expression was increased by 4-fold in ApoAIp compared to Cp cells (4.29 ± 0.64 versus 1.00 ± 0.11, *p* < 0.001) (Figure 2a). In good agreement, ApoAI protein expression in the total lysate was increased by 2-fold in ApoAIp cells above Cp (2.04 ± 0.14 versus 1.00 ± 0.04 for Cp, *p* < 0.001), while secreted ApoAI level in the culture medium of ApoAIp cells was 33% higher than in Cp (1.33 ± 0.04 versus 1.00 ± 0.03, *p* < 0.001) (Figure 2b,c). Interestingly, an increase of PON1 gene and protein expression, and its secretion was also observed in ApoAIp compared to Cp cells (1.30 ± 0.08 versus 1.00 ± 0.05, *p* < 0.05, 1.70 ± 0.13 versus 1.00 ± 0.08, *p* < 0.001, and 2.48 ± 0.35 versus 1.00 ± 0.03, respectively, *p* < 0.01) (Figure 2d–f).

### 3.2. Transcriptional Activation of Endogenous PON1 by CRISPR/dCas9 Technology Increases PON1 Expression and Secretion and Stimulates ApoAI Protein Production in Caco-2 Cells

After transfection of CRISPR/dCas9 PON1 plasmids, a 6-fold increase of PON1 gene expression was measured in PON1p compared to Cp cells (6.47 ± 0.66 versus 1.00 ± 0.05, *p* < 0.001). The protein expression of PON1 in total lysate from PON1p was 67% increased above Cp (1.67 ± 0.13 versus 1.00 ± 0.08, *p* < 0.001), while secreted PON1 levels in the medium of PON1p cells were increased by 3-fold compared to Cp cells (3.26 ± 0.76 versus 1.00 ± 0.03, *p* < 0.01) (Figure 2d–f). The protein expression of ApoAI was slightly stimulated by transfection of PON1p compared to Cp (1.46 ± 0.11 versus 1.00 ± 0.04 Cp, *p* < 0.01), but not enough to promote ApoAI secretion into the culture medium (Figure 2b,c).

### 3.3. Overexpression of Endogenous ApoAI and PON1 Stimulates ABCA1, SR-BI, and ABCG8 Protein Expression in Caco-2 Cells

**Figure 3:**
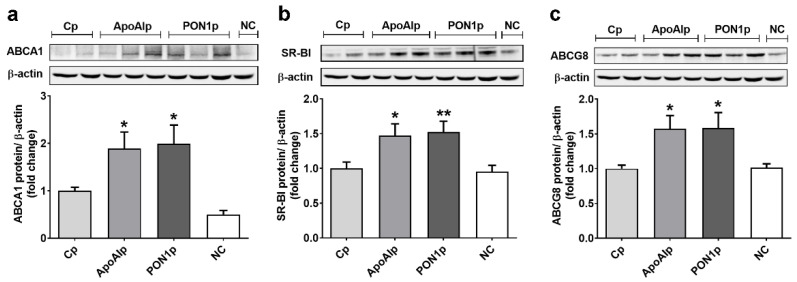
Overexpression of ApoAI and PON1 determines the increase of lipid transporters protein expression in Caco-2 cells. (**a**) ABCA1, (**b**) SR-BI, and (**c**) ABCG8 protein expression relative to β-actin (representative blot and densitometric analysis). Cp, control CRISPR activation plasmid; ApoAIp, CRISPR activation plasmids for ApoAI; PON1p, CRISPR activation plasmids for PON1; NC, negative control–untransfected Caco-2 cells. All data are expressed as fold change versus Cp and presented as mean ± SEM of 3 independent experiments. * *p* < 0.05, ** *p* < 0.01 vs. Cp.

We evaluated the effect of overexpressing ApoAI and PON1 on the expression of the ABCA1, scavenger receptor BI (SR-BI), and ABCG8 lipid transporters in Caco-2 cells. The results showed that the transcriptional activation of endogenous ApoAI or PON1 determines a 2-fold increase of ABCA1 protein expression as compared to Cp cells (1.89 ± 0.34 for ApoAIp and 1.99 ± 0.39 for PON1p versus 1.00 ± 0.07 for Cp, *p* < 0.05) (Figure 3a). The protein expression of SR-BI was upregulated by 50% in ApoAIp or PON1p compared to Cp cells (1.47 ± 0.17 for ApoAIp and 1.53 ± 0.15 for PON1p versus 1.00 ± 0.09 for Cp, *p* < 0.05 and *p* < 0.01, respectively) (Figure 3b). The expression of ABCG8 was increased by nearly 60% in ApoAIp and PON1p cells, as compared to Cp cells (1.57 ± 0.19 for ApoAIp and 1.58 ± 0.22 for PON1p versus 1.00 ± 0.05 for Cp, *p* < 0.05) (Figure 3c).

### 3.4. Overexpression of Endogenous ApoAI and PON-1 Upregulates the Expression of Lipid Related Transcription Factors in Caco-2 Cells

**Figure 4:**
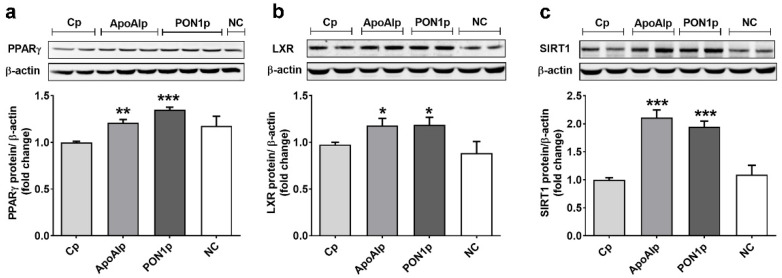
Overexpression of ApoAI and PON1 increases the protein expression of transcription factors involved in lipid transporters regulation in Caco-2 cells. (**a**) PPARγ, (**b**) LXR, and (**c**) SIRT1 protein expression relative to β-actin (representative blot and densitometric analysis). Cp, control CRISPR activation plasmid; ApoAIp, CRISPR activation plasmids for ApoAI; PON1p, CRISPR activation plasmids for PON1; NC, negative control–untransfected Caco-2 cells. All data are expressed as fold change versus Cp and presented as mean ± SEM of 3 independent experiments. * *p* < 0.05, ** *p* < 0.01, *** *p* < 0.001 vs. Cp.

The protein expression of PPARγ was increased by over 20% in ApoAIp or PON1p compared to Cp cells (1.21 ± 0.03 for ApoAIp and 1.35 ± 0.03 for PON1p versus 1.00 ± 0.01 for Cp, *p* < 0.001 and *p* < 0.001, respectively) (Figure 4a). Additionally, LXR protein expression was increased by 18% in ApoAIp or PON1p compared to Cp cells (1.18 ± 0.08 for ApoAIp and PON1p versus 1.00 ± 0.03 for Cp, *p* < 0.05 for both) (Figure 4b). SIRT1 expression was 2-fold stimulated in ApoAIp and PON1p compared to Cp cells (2.11 ± 0.13 for ApoAIp and 1.94 ± 0.10 for PON1p versus 1.00 ± 0.04 for Cp, *p* < 0.001 for both ApoAIp and PON1p) (Figure 4c).

### 3.5. Conditioned Media from Caco-2 Cells Overexpressing Endogenous ApoAI or PON1 Have Anti-Inflammatory and Antioxidant Effects on TNFα-Activated EC

**Figure 5:**
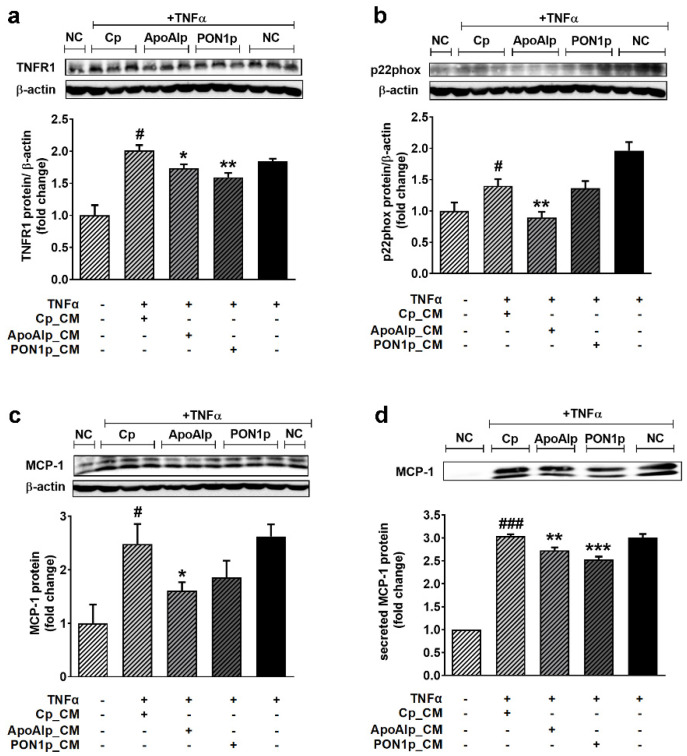
Conditioned media from Caco-2 overexpressing ApoAI and PON1 decrease inflammation in TNF-α activated EC. (**a**) TNFR1, (**b**) p22phox, and (**c**) MCP-1 protein expression relative to β-actin (representative blot and densitometric analysis). (**d**) Secreted MCP-1 in the culture medium relative to total cell protein (representative blots and densitometric analysis). Cp, control CRISPR activation plasmid; ApoAIp, CRISPR activation plasmids for ApoAI; PON1p, CRISPR activation plasmids for PON1; CM, conditioned media; NC, negative control–EC incubated with CM from untransfected Caco-2 cells. All data are expressed as fold change versus Cp and presented as mean ± SEM of 3 independent experiments. # *p* < 0.05, ### *p* < 0.001 vs. NC; * *p* < 0.05, ** *p* < 0.01, *** *p* < 0.001 vs. Cp_CM.

EC function is highly important for the homeostasis of the vascular bed. Thus, the potential of the CM from Caco2 cells overexpressing ApoAI or PON1 to reduce the TNFα-induced EC dysfunction was next evaluated by measuring the protein expression of pro-inflammatory proteins tumor necrosis factor receptor 1 (TNFR1) and MCP-1, and of the regulatory subunit of NADPH oxidase, p22phox. The obtained results showed that the CM enriched in secreted ApoAI or PON1 decrease by over 25% the protein expression of TNFR1 in TNFα-activated EC, as compared to CM from Cp cells (1.73 ± 0.06 for ApoA1p and 1.59 ± 0.07 for PON1p versus 2.00 ± 0.08 for Cp, *p* < 0.05 and *p* < 0.01, respectively) (Figure 5a). CM from ApoAIp cells decreased by 35% the protein expression of MCP-1 in the total lysate of TNFα-activated EC compared to CM from Cp cells (1.61 ± 0.16 versus 2.48 ± 0.37, *p* < 0.05). The levels of MCP-1 protein expression showed a 25% decrease in TNFα-activated EC treated with CM of PON1p cells, but this did not reach statistical significance (Figure 5c). The level of secreted MCP-1 in EC media was over 25% decreased by the CM from ApoAIp or PON1p compared to Cp cells (2.73 ± 0.06 for ApoAIp, and 2.53 ± 0.06 for PON1p versus 3.03 ± 0.04 for Cp, *p* < 0.01 and *p* < 0.001, respectively) (Figure 5d). Compared to Cp cell medium, the CM enriched in secreted ApoAI decreased by 50% the protein expression of NADPH oxidase regulatory subunit p22phox in TNFα-exposed EC (0.89 ± 0.09 versus 1.40 ± 0.11, *p* < 0.01) (Figure 5b).

## 4. Discussion

A challenging therapeutic strategy against the atherogenic process is the restoration of HDL function, which involves improving the quantity and quality of key HDL proteins. Several studies have been performed to evaluate the beneficial effects of exogenously administrated recombinant ApoAI in CVD patients, as well as reassembled or mimetic HDL particles [26,30]. These studies have shown promising findings, including increased ApoAI plasma levels and enhanced HDL cholesterol efflux capacity, but ApoAI-containing agents failed to demonstrate clinical benefits [31,32]. Beside this, exogenously administrated ApoAI may fail to exert its beneficial effects due to the alterations it can suffer from the oxidative stress known to occur in CVD conditions [26]. The hypothesis of our study was that activating the transcription of endogenous ApoAI or PON1 in enterocytes will enable the cells to synthetize and secrete these proteins in elevated amounts to protect EC against excessive inflammation.

The novel findings of our study are that the transcription activation of endogenous ApoAI and PON1 in Caco-2 enterocytes by using the CRISPR/dCas9 system induces (i) significant increase of their gene and protein expression, as well as their secretion in the culture medium; (ii) additional stimulation of the endogenous transporters ABCA1 and SR-BI which are implicated in HDL maturation and function; (iii) upregulation of the expression of ABCG8 transporter involved in the cholesterol efflux from enterocytes; (iv) positive regulation of the transcriptional factors LXRs, PPARγ, and SIRT1 that control lipid metabolism. CM from Caco-2 cells overexpressing ApoAI and PON1 attenuate the TNFα-induced inflammatory stress in EC by decreasing TNFR1, MCP-1, and p22phox protein expression.

The small intestine is the second most important organ for the HDL biogenesis. Enterocytes synthesize the major HDL proteins ApoAI and PON1 and contribute to HDL formation through the interaction of the secreted lipid-poor ApoAI and the ABCA1 membrane-transporter, by which ApoAI becomes lipidated, generating preβ-HDL [33,34]. We have previously reported that ApoAI, PON1, and ABCA1 levels in the small intestine are inversely correlated with the severity of atherosclerosis [35,36]. The downregulation of key HDL proteins in the small intestine in hyperlipidemia is due to decreased levels of their transcriptional activators PPARγ, LXRs, and SIRT1, as we have shown before [35,36]. Moreover, we demonstrated that a high-fat diet induces oxidized-ApoAI production in the small intestine [36]. Until now, enterocytes had not been evaluated as potential target cells for the overproduction of major HDL proteins. In the present study we show for the first time that transcriptional activation of endogenous ApoAI and PON1 in enterocytes by using the CRISPR/dCas9 system generates a significant increase of their secreted levels. It is notable that PON1 is secreted to a greater extent compared to ApoAI, and this could ensure an antioxidant protection for the nearby lipids and proteins. It is also noteworthy that ApoAI and PON1 stimulate the synthesis of each other. Moreover, in both types of transfected cells, the protein expression of the ABCA1 transporter was upregulated. The CRISPR/dCas9 technology is known as a very accurate and efficient gene-editing system, with generally minimal off-target effects [37]. Therefore, an explanation for the mutual stimulation of ApoAI and PON1, and for their effect on ABCA1 protein expression could be the increase of the levels of their transcriptional activators PPARγ, LXRs, and SIRT1. Our results are supported by the group of Wang et al. who published data showing that human-apoAI treatment upregulates PPARγ expression in THP-1 macrophages [38]. Moreover, very recently published results show that lentiviral-induced human-PON1 overexpression in the liver of SR-BI deficient mice upregulates LXR and its downstream genes, including ApoAI and ABCA1 [39].

The role of SR-B1 in enterocytes is still under debate. It was reported to mediate cholesterol transport across the enterocyte plasma membrane, as well as the absorption of lipophilic antioxidants from the intestinal lumen to the blood [22]. Our results show that overexpression of endogenous ApoAI or PON1 in enterocytes induces an increase of the SR-BI protein level. The gene expression of SR-BI is controlled both by PPARγ and LXRs [22]. Their upregulation by ApoAI or PON1 could be responsible for the increase of SR-BI protein expression, which might have a positive impact on the uptake of lipophilic antioxidants from the intestinal lumen.

Another important process that takes place in the small intestine is the trans-intestinal cholesterol efflux (TICE) by which cholesterol is directly excreted from enterocytes into the intestinal lumen [40]. The heterodimer ABCG5/8 transporters located in the apical membrane of enterocytes are responsible for the TICE [41], and their transcription is controlled by LXRs. We have previously shown that a high-fat diet decreases LXR expression and the protein expression of ABCG8 in the small intestine of hyperlipidemic hamsters [36]. The present results show for the first time that the overexpression of endogenous ApoAI or PON1 induces an increase of ABCG8 protein expression, and thus could facilitate the removal of excess cholesterol from enterocytes.

**Figure 6:**
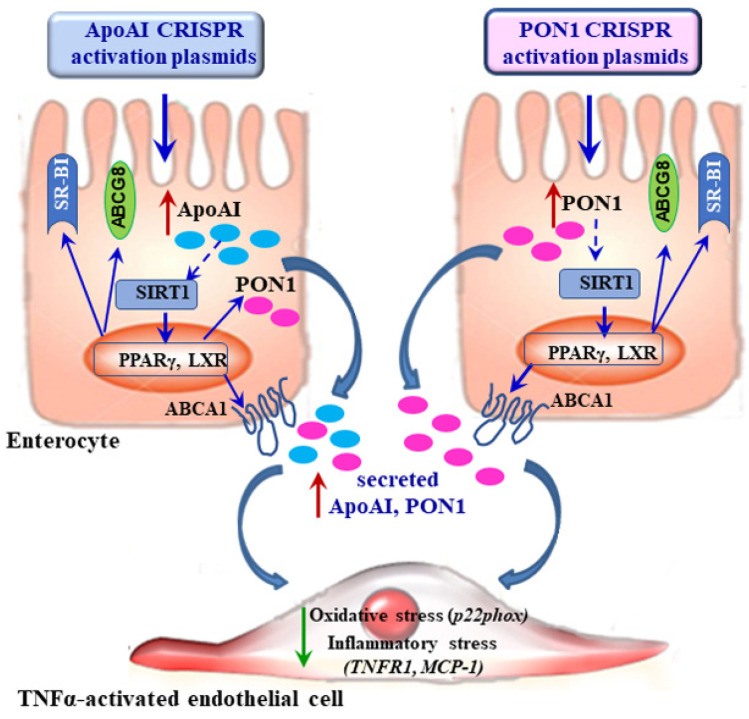
Schematic representation of the benefic effects induced by the transcriptional activation of the endogenous apoAI or PON1 in enterocytes. Transfection of Caco-2 cells with CRISPR/dCas9 activation plasmids for apoAI or PON1 increases their gene and protein expression, as well as their secretion in the culture medium. Overexpressed apoAI and PON1 exert a positive feedback regulation on their transcription factors PPARγ, LXRs, and SIRT1. In turn, these transcription factors upregulate the lipid transporters ABCA1, ABCG8, and SR-BI which are known to be anti-atherogenic. The apoAI and PON1 secreted by the transfected enterocytes in the culture medium are able to attenuate the TNFα-induced inflammatory and oxidative stress in EC, by decreasing TNFR1, MCP-1, and p22phox levels.

Atherosclerosis is an inflammatory disease and a key event in the atheroma formation is the EC dysfunction induced by various risk factors [5,42]. The TNFα signaling pathway is a central pro-inflammatory pathway involved in atherosclerosis progression [43]. Functional HDL are able to protect EC, reversing the pro-inflammatory status of activated EC [44]. Our results show that the endogenous ApoAI and PON1 secreted by the transfected enterocytes exert anti-inflammatory effects in TNFα-activated EC by reducing the protein expression of TNFR1 and the secreted MCP-1 in the culture medium (Figure 6). The pronounced antioxidant effect of the conditioned medium from ApoAIp-transfected enterocytes exerted on the protein expression of NADPHox subunit p22phox can be explained by the higher content of secreted ApoAI and PON1 compared to the conditioned medium from PON1-transfected enterocytes.

The obtained results are encouraging for the use of enterocytes as a promising source of functional HDL. Nevertheless, the present study has few limitations. First, more in-depth studies are needed to explain the mechanisms by which ApoAI or PON1 induce the upregulation of SIRT1 and of LXR and PPARΥ transcription factors. Second, the translation of our results from the in vitro study to an animal model is necessary to validate this method by which the enterocytes can function as source of functional HDL.

It is generally accepted that improvement of HDL function rather than increase of HDL-cholesterol level is more beneficial for the CVD patients. Improvement of HDL function involves the increase of the level and quality of their major anti-atherogenic proteins, such as apoAI and PON1. ApoAI is a protein with multiple beneficial effects. Using different experimental models, it was reported that the increase of ApoI reduces the incidence of heart failure, improves glycemic control, presents anti-tumor activities, and acts as a neuro-protector, reducing the deposition of amyloid β and neuroinflammation [45]. The pre-clinical studies show that ApoAI treatment promotes the regression of atherosclerotic lesions, especially in the early stages [46], and reduces vascular inflammation [47,48]. Unfortunately, the positive outcomes of the preclinical observations have not been observed in randomized clinical trials in patients with acute coronary syndrome (ACS), in which reassembled HDL containing ApoAI Milano or wild-type ApoAI were infused weekly [49,50]. These negative results were attributed to the post-translational modification of ApoAI in the highly pro-inflammatory and pro-oxidant milieu due to ACS condition. Published data show that in a pro-oxidant state, ApoAI can become oxidized and even cleaved by the granule-associate proteases liberated by the mast cells [51]. These modifications determine the formation of a dysfunctional ApoAI, with no antioxidant and anti-inflammatory properties. In this context, our study addresses two main relevant clinical questions: (1) can we consider enterocytes a source of good quality ApoAI? (2) can we assure a protective milieu to conserve ApoAI functionality? Our results indicate that overexpression of ApoAI in Caco-2 cells renders a functional protein, able to ameliorate EC dysfunction. Very important, our study demonstrates that induced overexpression of ApoAI by using the CRISPR/dCas9 technology determines the concomitant upregulation of PON1, which can be considered a result with therapeutic consequences. This is due to the fact that PON1, a protein with antioxidant properties can generate a friendly medium for ApoAI, protecting its structure and function. Additionally, the fact that the conditioned medium of Caco-2 cells overexpressing ApoAI or PON1 is able to protect EC against inflammatory stress is very important, being well known that EC dysfunction is an important step in the inception and progress of atherosclerosis and many other pathologies.

Preliminary results of clinical studies show that CRISPR/Cas9 technology has been successfully used to treat neurological disorders [52]. Our results could be translated into practical applications and used in the future for the treatment of CVD patients.

## 5. Conclusions

In the present study we show that the transcriptional activation of the endogenous ApoAI and PON1 in enterocytes by using the CRISPR/dCas9 system is a realistic approach to stimulate their secretion in order to improve HDL function. The ApoAI and PON1 overexpressed by enterocytes are able to stimulate transporters involved in cholesterol efflux and to reduce the inflammatory stress in activated EC.

## Figures and Tables

**Table 1:** Sequence of primers used for Real-Time PCR analysis.

Gene	GeneBank Accession Number	Sequences of Oligonucleotide Primers
ApoAI	NM_000039.2	FW: 5′-CCCTGGGATCGAGTGAAGGA-3′ RV: 5′-CTGGGACACATAGTCTCTGCC-3′
PON1	NM_000446.5	FW: 5′-CTATGACTCAGAGAATCCTCCTGCATCAG-3′ RV: 5′-CATGGGTGCAAATCGGTCTGTTAGAGC-3′
β-actin	NM_001101.3	FW: 5′-GTCTTCCCCTCCATCGT-3′ RV: 5′-CGTCGCCCACATAGGAAT-3′

ApoAI, apolipoprotein AI; PON1, paraoxonase 1.

**Table 2:** Antibodies used for Western blot analysis.

Antibody	Specificity	Catalogue	Dilution	Source
Mouse mAb	Human ApoAI	sc-69755	1:600	Santa Cruz
Mouse mAb	Human PON1	ab24261	1:500	Abcam
Rabbit pAb	Human ABCA1	NB400105	1:500	Novus Biologicals
Rabbit pAb	Human ABCG8	sc-30111	1:500	Santa Cruz
Mouse mAb	Human LXR	sc-377260	1:500	Santa Cruz
Rabbit pAb	Human PPAR-γ	sc-7196	1:500	Santa Cruz
Mouse mAb	Human SIRT-1	NBP1-51641	1:1000	Novus Biologicals
Rabbit mAb	Human SR-BI	ab217318	1:2000	Abcam
Mouse mAb	Human TNFR1	sc-8436	1:400	Santa Cruz
Rabbit pAb	Human MCP-1	ab9669	1:1000	Abcam
Rabbit pAb	Human p22phox	ab75941	1:1000	Abcam
Mouse mAb	Human β-actin	sc-47778	1:4000	Santa Cruz
	Rabbit Anti-Mouse IgG H&L (HRP)	ab6728	1:10,000	Abcam
	Rabbit Anti-Goat IgG H&L (HRP)	ab6741	1:10,000	Abcam
	Goat Anti-Rabbit IgG H&L (HRP)	ab6721	1:10,000	Abcam

ApoAI, apolipoprotein AI; PON1, paraoxonase 1; ABCA1/G8, ATP binding cassette A1 and G8 transporters; LXR, liver X receptors; PPARγ, peroxisome proliferator-activated receptor γ; SIRT1, sirtuin-1; SR-BI, scavenger receptor BI; TNFR1, tumor necrosis factor receptor 1; MCP-1, monocyte chemoattractant protein 1; p22phox, p22phox subunit of NADPH oxidase; mAb, monoclonal antibody; pAb, polyclonal antibody^.^

## Data Availability

Data available on request.
